# *N*′-(3,5-Dichloro-2-hydroxy­benzyl­idene)-3-methoxy­benzohydrazide methanol solvate

**DOI:** 10.1107/S160053680801235X

**Published:** 2008-04-30

**Authors:** Chun-Hua Ling, Yan-Bin Chen, Jian-An Huang, Cheng Ji, Peng Liu

**Affiliations:** a Respiratory Department, The First Affiliated Hospital of Soochow University, Suzhou Jiangsu 215006, People’s Republic of China; b College of Chemistry, Liaoning Teacher University, Dalian 116029, People’s Republic of China

## Abstract

In the title compound, C_15_H_12_Cl_2_N_2_O_3_·CH_3_OH, the Schiff base mol­ecule is nearly planar, with a dihedral angle of 4.5 (2)° between the two benzene rings. An intra­molecular O—H⋯N hydrogen bond is observed. The methanol solvent mol­ecule is linked to the Schiff base mol­ecule through inter­molecular N—H⋯O and O—H⋯O hydrogen bonds.

## Related literature

For the synthesis of Schiff base compounds, see: Herrick *et al.* (2008 ▶); Suresh *et al.* (2007 ▶); Liu *et al.* (2007 ▶). For the background on biological activities, see: Bhandari *et al.* (2008 ▶); Sinha *et al.* (2008 ▶); Sun *et al.* (2008 ▶). For related structures, see: Wang *et al.* (2008 ▶); Tang (2008*a* ▶,*b* ▶); Yang & Zheng (2007 ▶).
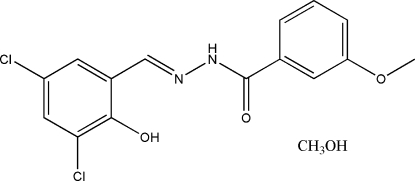


## Experimental

### 

#### Crystal data

C_15_H_12_Cl_2_N_2_O_3_·CH_4_O
*M* *_r_* = 371.21
Triclinic, 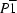

*a* = 7.742 (3) Å
*b* = 9.070 (3) Å
*c* = 12.296 (4) Å
α = 92.422 (5)°
β = 98.948 (5)°
γ = 96.954 (5)°
*V* = 845.0 (5) Å^3^
*Z* = 2
Mo *K*α radiation
μ = 0.41 mm^−1^
*T* = 298 (2) K
0.27 × 0.23 × 0.20 mm

#### Data collection

Bruker SMART CCD area-detector diffractometer
Absorption correction: multi-scan (*SADABS*; Sheldrick, 1996 ▶) *T*_min_ = 0.898, *T*_max_ = 0.923
6888 measured reflections
3452 independent reflections
2253 reflections with *I* > 2σ(*I*)
*R*_int_ = 0.029

#### Refinement

*R*[*F* ^2^ > 2σ(*F* ^2^)] = 0.051
*wR*(*F* ^2^) = 0.131
*S* = 1.04
3452 reflections
224 parameters
1 restraint
H atoms treated by a mixture of independent and constrained refinement
Δρ_max_ = 0.27 e Å^−3^
Δρ_min_ = −0.24 e Å^−3^

### 

Data collection: *SMART* (Bruker, 2002 ▶); cell refinement: *SAINT* (Bruker, 2002 ▶); data reduction: *SAINT*; program(s) used to solve structure: *SHELXS97* (Sheldrick, 2008 ▶); program(s) used to refine structure: *SHELXL97* (Sheldrick, 2008 ▶); molecular graphics: *SHELXTL* (Sheldrick, 2008 ▶); software used to prepare material for publication: *SHELXL97*.

## Supplementary Material

Crystal structure: contains datablocks global, I. DOI: 10.1107/S160053680801235X/ci2593sup1.cif

Structure factors: contains datablocks I. DOI: 10.1107/S160053680801235X/ci2593Isup2.hkl

Additional supplementary materials:  crystallographic information; 3D view; checkCIF report

## Figures and Tables

**Table 1:** Hydrogen-bond geometry (Å, °)

*D*—H⋯*A*	*D*—H	H⋯*A*	*D*⋯*A*	*D*—H⋯*A*
N2—H2⋯O4^i^	0.899 (10)	1.997 (12)	2.881 (3)	167 (3)
O4—H4⋯O2	0.82	2.35	2.989 (3)	135
O4—H4⋯O2^ii^	0.82	2.34	3.023 (3)	141
O1—H1⋯N1	0.82	1.84	2.557 (3)	145

Symmetry codes: (i) 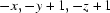; (ii) 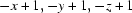.

**Fig. 1.**
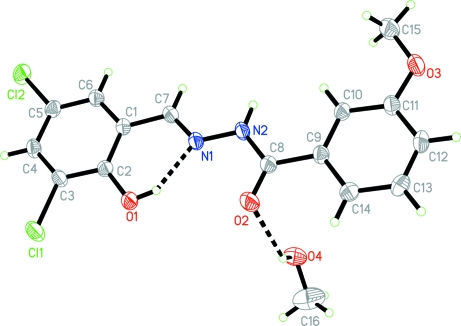
The molecular structure of the title compound, showing 30% probability displacement ellipsoids. Intramolecular hydrogen bonds are shown as dashed lines.

**Fig. 2.**
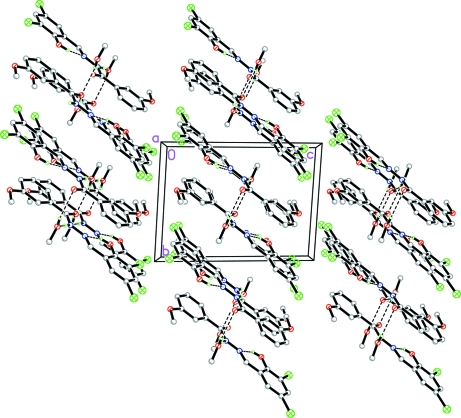
The molecular packing of the title compound, viewed along the a axis. Intermolecular hydrogen bonds are shown as dashed lines. H atoms not involved in the hydrogen bonds have been omitted for clarity.

## supplementary crystallographic information

### Comment

Schiff base compounds can be easily synthesized from the reaction of aldehydes with primary amines (Herrick *et al.*, 2008; Suresh *et al.*, 2007; Liu *et al.*, 2007). These compounds show interesting biological activities, especially antimicrobial activities (Bhandari *et al.*, 2008; Sinha *et al.*, 2008; Sun *et al.*, 2008). Recently, the crystal structures of a few Schiff base compounds obtained from the derivatives of salicylaldehyde with benzohydrazide have been reported (Wang *et al.*, 2008; Tang, 2008*a*,b; Yang & Zheng, 2007). We report here the crystal structure of a new Schiff base compound, derived from 3,5-dichlorosalicylaldehyde and 3-methoxybenzohydrazide.

The asymmetric unit consists of a Schiff base molecule and a methanol molecule of crystallization (Fig. 1). The Schiff base molecule is nearly planar, with a maximum deviation of 0.133 (1) Å for atom Cl1. The dihedral angle between the two benzene rings is 4.5 (2)°. An intramolecular O—H···N hydrogen bond is observed in the Schiff base molecule. The methanol molecule of crystallization is linked to the Schiff base molecule through intermolecular N—H···O and O—H···O hydrogen bonds (Table 1 and Fig.2).

### Experimental

3,5-Dichlorosalicylaldehyde (0.1 mmol, 19.0 mg) and 3-methoxybenzohydrazide (0.1 mmol, 16.6 mg) were dissolved in methanol (20 ml). The mixture was stirred at room temperature to give a clear yellow solution. Yellow block-shaped crystals were formed after a week.

### Refinement

Atom H2 was located in a difference Fourier map and refined isotropically, with the N2—H2 distance restrained to 0.90 (1) Å, and with *U*_iso_(H) set to 0.08 Å^2^. All other H atoms were constrained to idealized geometries, with C–H = 0.93–0.96 Å, O–H = 0.82 Å, and with *U*_iso_(H) = 1.2*U*_eq_(C) and 1.5*U*_eq_(O and methyl C). A rotating group model was used for the methyl and hydroxyl groups.

### Crystal data

**Table:**

C_15_H_12_Cl_2_N_2_O_3_·CH_4_O	*Z* = 2
*M**_r_* = 371.21	*F*_000_ = 384
Triclinic, *P*1	*D*_x_ = 1.459 Mg m^−^^3^
Hall symbol: -P 1	Mo *K*α radiation λ = 0.71073 Å
*a* = 7.742 (3) Å	Cell parameters from 1304 reflections
*b* = 9.070 (3) Å	θ = 2.4–24.5º
*c* = 12.296 (4) Å	µ = 0.41 mm^−^^1^
α = 92.422 (5)º	*T* = 298 (2) K
β = 98.948 (5)º	Block, yellow
γ = 96.954 (5)º	0.27 × 0.23 × 0.20 mm
*V* = 845.0 (5) Å^3^	

### Data collection

**Table:**

Bruker SMART CCD area-detector diffractometer	3452 independent reflections
Radiation source: fine-focus sealed tube	2253 reflections with *I* > 2σ(*I*)
Monochromator: graphite	*R*_int_ = 0.029
*T* = 298(2) K	θ_max_ = 26.5º
ω scans	θ_min_ = 1.7º
Absorption correction: multi-scan(SADABS; Sheldrick, 1996)	*h* = −9→9
*T*_min_ = 0.898, *T*_max_ = 0.923	*k* = −11→11
6888 measured reflections	*l* = −15→15

### Refinement

**Table:**

Refinement on *F*^2^	Secondary atom site location: difference Fourier map
Least-squares matrix: full	Hydrogen site location: inferred from neighbouring sites
*R*[*F*^2^ > 2σ(*F*^2^)] = 0.051	H atoms treated by a mixture of independent and constrained refinement
*wR*(*F*^2^) = 0.131	*w* = 1/[σ^2^(*F*_o_^2^) + (0.0565*P*)^2^] where *P* = (*F*_o_^2^ + 2*F*_c_^2^)/3
*S* = 1.04	(Δ/σ)_max_ = 0.001
3452 reflections	Δρ_max_ = 0.27 e Å^−^^3^
224 parameters	Δρ_min_ = −0.24 e Å^−^^3^
1 restraint	Extinction correction: none
Primary atom site location: structure-invariant direct methods	

### Special details

**Table:**

Geometry. All e.s.d.'s (except the e.s.d. in the dihedral angle between two l.s. planes) are estimated using the full covariance matrix. The cell e.s.d.'s are taken into account individually in the estimation of e.s.d.'s in distances, angles and torsion angles; correlations between e.s.d.'s in cell parameters are only used when they are defined by crystal symmetry. An approximate (isotropic) treatment of cell e.s.d.'s is used for estimating e.s.d.'s involving l.s. planes.
Refinement. Refinement of *F*^2^ against ALL reflections. The weighted *R*-factor *wR* and goodness of fit *S* are based on *F*^2^, conventional *R*-factors *R* are based on *F*, with *F* set to zero for negative *F*^2^. The threshold expression of *F*^2^ > σ(*F*^2^) is used only for calculating *R*-factors(gt) *etc*. and is not relevant to the choice of reflections for refinement. *R*-factors based on *F*^2^ are statistically about twice as large as those based on *F*, and *R*- factors based on ALL data will be even larger.

### Fractional atomic coordinates and isotropic or equivalent isotropic displacement parameters (Å^2^)

**Table:**

	*x*	*y*	*z*	*U*_iso_*/*U*_eq_	
Cl1	0.29231 (9)	−0.03639 (9)	0.10217 (6)	0.0659 (3)	
Cl2	−0.37695 (9)	−0.28076 (8)	0.09465 (6)	0.0636 (3)	
O1	0.2383 (2)	0.14041 (19)	0.29003 (15)	0.0532 (5)	
H1	0.2219	0.1852	0.3461	0.080*	
O2	0.3328 (2)	0.3979 (2)	0.51936 (17)	0.0729 (6)	
O3	−0.0405 (3)	0.5968 (2)	0.88407 (15)	0.0659 (6)	
O4	0.3034 (2)	0.6965 (2)	0.42945 (18)	0.0677 (6)	
H4	0.3679	0.6328	0.4458	0.102*	
N1	0.0576 (3)	0.2173 (2)	0.43529 (16)	0.0432 (5)	
N2	0.0515 (3)	0.3093 (2)	0.52560 (17)	0.0455 (5)	
C1	−0.0641 (3)	0.0367 (3)	0.2932 (2)	0.0408 (6)	
C2	0.0927 (3)	0.0466 (3)	0.2486 (2)	0.0406 (6)	
C3	0.0983 (3)	−0.0464 (3)	0.1565 (2)	0.0432 (6)	
C4	−0.0443 (3)	−0.1470 (3)	0.1091 (2)	0.0474 (6)	
H4A	−0.0375	−0.2083	0.0477	0.057*	
C5	−0.1966 (3)	−0.1549 (3)	0.1541 (2)	0.0448 (6)	
C6	−0.2080 (3)	−0.0639 (3)	0.2446 (2)	0.0460 (6)	
H6	−0.3127	−0.0699	0.2733	0.055*	
C7	−0.0786 (3)	0.1315 (3)	0.3889 (2)	0.0483 (7)	
H7	−0.1848	0.1295	0.4156	0.058*	
C8	0.2044 (3)	0.3995 (3)	0.5653 (2)	0.0438 (6)	
C9	0.2074 (3)	0.4983 (3)	0.66579 (19)	0.0419 (6)	
C10	0.0683 (3)	0.4953 (3)	0.7255 (2)	0.0422 (6)	
H10	−0.0351	0.4307	0.7029	0.051*	
C11	0.0864 (3)	0.5898 (3)	0.8188 (2)	0.0469 (6)	
C12	0.2406 (4)	0.6849 (3)	0.8525 (2)	0.0557 (7)	
H12	0.2523	0.7472	0.9159	0.067*	
C13	0.3750 (4)	0.6877 (3)	0.7933 (2)	0.0596 (8)	
H13	0.4778	0.7530	0.8161	0.072*	
C14	0.3607 (3)	0.5946 (3)	0.6997 (2)	0.0505 (7)	
H14	0.4535	0.5966	0.6598	0.061*	
C15	−0.2031 (4)	0.5035 (4)	0.8545 (3)	0.0710 (9)	
H15A	−0.2575	0.5241	0.7821	0.107*	
H15B	−0.2796	0.5218	0.9066	0.107*	
H15C	−0.1824	0.4012	0.8547	0.107*	
C16	0.3991 (5)	0.8175 (4)	0.3948 (4)	0.1132 (15)	
H16A	0.3265	0.8956	0.3822	0.170*	
H16B	0.4388	0.7899	0.3274	0.170*	
H16C	0.4992	0.8515	0.4503	0.170*	
H2	−0.052 (2)	0.307 (3)	0.551 (2)	0.080*	

### Atomic displacement parameters (Å^2^)

**Table:**

	*U*^11^	*U*^22^	*U*^33^	*U*^12^	*U*^13^	*U*^23^
Cl1	0.0460 (4)	0.0854 (6)	0.0652 (5)	−0.0006 (4)	0.0214 (3)	−0.0269 (4)
Cl2	0.0477 (4)	0.0620 (5)	0.0732 (5)	−0.0078 (3)	0.0037 (3)	−0.0223 (4)
O1	0.0428 (10)	0.0569 (11)	0.0563 (12)	−0.0079 (9)	0.0142 (8)	−0.0200 (9)
O2	0.0505 (12)	0.0888 (15)	0.0777 (15)	−0.0132 (11)	0.0315 (11)	−0.0309 (12)
O3	0.0605 (12)	0.0825 (14)	0.0529 (12)	−0.0024 (11)	0.0200 (10)	−0.0229 (10)
O4	0.0470 (12)	0.0815 (15)	0.0784 (15)	0.0085 (10)	0.0202 (11)	0.0104 (12)
N1	0.0435 (12)	0.0456 (12)	0.0397 (12)	0.0026 (10)	0.0100 (9)	−0.0096 (10)
N2	0.0402 (12)	0.0526 (13)	0.0425 (12)	0.0005 (10)	0.0118 (10)	−0.0158 (10)
C1	0.0434 (14)	0.0383 (13)	0.0404 (14)	0.0037 (11)	0.0092 (11)	−0.0040 (11)
C2	0.0353 (13)	0.0410 (13)	0.0438 (15)	0.0014 (11)	0.0064 (11)	−0.0044 (11)
C3	0.0396 (14)	0.0501 (15)	0.0410 (14)	0.0070 (11)	0.0121 (11)	−0.0071 (12)
C4	0.0487 (15)	0.0480 (15)	0.0437 (15)	0.0070 (12)	0.0053 (12)	−0.0125 (12)
C5	0.0385 (14)	0.0426 (14)	0.0492 (16)	−0.0004 (11)	0.0013 (11)	−0.0053 (12)
C6	0.0401 (14)	0.0469 (15)	0.0502 (16)	0.0013 (11)	0.0104 (12)	−0.0080 (12)
C7	0.0466 (15)	0.0508 (15)	0.0485 (16)	0.0016 (13)	0.0175 (13)	−0.0084 (13)
C8	0.0391 (14)	0.0456 (15)	0.0461 (15)	0.0019 (12)	0.0098 (12)	−0.0052 (12)
C9	0.0404 (14)	0.0446 (14)	0.0388 (14)	0.0048 (11)	0.0030 (11)	−0.0046 (11)
C10	0.0357 (13)	0.0455 (14)	0.0418 (14)	−0.0018 (11)	0.0031 (11)	−0.0072 (11)
C11	0.0487 (15)	0.0491 (15)	0.0419 (15)	0.0061 (12)	0.0063 (12)	−0.0059 (12)
C12	0.0621 (18)	0.0551 (17)	0.0444 (16)	−0.0004 (14)	0.0028 (14)	−0.0153 (13)
C13	0.0510 (17)	0.0602 (18)	0.0581 (19)	−0.0117 (14)	−0.0029 (14)	−0.0109 (15)
C14	0.0427 (15)	0.0538 (16)	0.0524 (17)	−0.0013 (12)	0.0076 (12)	−0.0038 (13)
C15	0.0575 (19)	0.083 (2)	0.075 (2)	0.0027 (17)	0.0269 (16)	−0.0150 (18)
C16	0.090 (3)	0.103 (3)	0.148 (4)	−0.001 (2)	0.025 (3)	0.045 (3)

### Geometric parameters (Å, °)

**Table:**

Cl1—C3	1.730 (2)	C5—C6	1.377 (3)
Cl2—C5	1.736 (2)	C6—H6	0.93
O1—C2	1.344 (3)	C7—H7	0.93
O1—H1	0.82	C8—C9	1.490 (3)
O2—C8	1.219 (3)	C9—C14	1.382 (3)
O3—C11	1.367 (3)	C9—C10	1.393 (3)
O3—C15	1.417 (3)	C10—C11	1.381 (3)
O4—C16	1.369 (4)	C10—H10	0.93
O4—H4	0.82	C11—C12	1.381 (3)
N1—C7	1.273 (3)	C12—C13	1.358 (4)
N1—N2	1.371 (3)	C12—H12	0.93
N2—C8	1.364 (3)	C13—C14	1.381 (4)
N2—H2	0.899 (10)	C13—H13	0.93
C1—C6	1.390 (3)	C14—H14	0.93
C1—C2	1.402 (3)	C15—H15A	0.96
C1—C7	1.455 (3)	C15—H15B	0.96
C2—C3	1.393 (3)	C15—H15C	0.96
C3—C4	1.379 (3)	C16—H16A	0.96
C4—C5	1.374 (3)	C16—H16B	0.96
C4—H4A	0.93	C16—H16C	0.96
			
C2—O1—H1	109.5	C14—C9—C10	120.3 (2)
C11—O3—C15	118.7 (2)	C14—C9—C8	116.0 (2)
C16—O4—H4	109.5	C10—C9—C8	123.7 (2)
C7—N1—N2	120.9 (2)	C11—C10—C9	119.0 (2)
C8—N2—N1	115.25 (19)	C11—C10—H10	120.5
C8—N2—H2	127.0 (19)	C9—C10—H10	120.5
N1—N2—H2	117.7 (19)	O3—C11—C10	124.3 (2)
C6—C1—C2	119.6 (2)	O3—C11—C12	115.3 (2)
C6—C1—C7	119.6 (2)	C10—C11—C12	120.4 (2)
C2—C1—C7	120.8 (2)	C13—C12—C11	120.3 (2)
O1—C2—C3	118.0 (2)	C13—C12—H12	119.9
O1—C2—C1	123.7 (2)	C11—C12—H12	119.9
C3—C2—C1	118.2 (2)	C12—C13—C14	120.7 (2)
C4—C3—C2	122.0 (2)	C12—C13—H13	119.7
C4—C3—Cl1	119.52 (18)	C14—C13—H13	119.7
C2—C3—Cl1	118.44 (18)	C13—C14—C9	119.5 (2)
C5—C4—C3	118.8 (2)	C13—C14—H14	120.3
C5—C4—H4A	120.6	C9—C14—H14	120.3
C3—C4—H4A	120.6	O3—C15—H15A	109.5
C4—C5—C6	120.9 (2)	O3—C15—H15B	109.5
C4—C5—Cl2	119.08 (19)	H15A—C15—H15B	109.5
C6—C5—Cl2	119.97 (19)	O3—C15—H15C	109.5
C5—C6—C1	120.4 (2)	H15A—C15—H15C	109.5
C5—C6—H6	119.8	H15B—C15—H15C	109.5
C1—C6—H6	119.8	O4—C16—H16A	109.5
N1—C7—C1	118.4 (2)	O4—C16—H16B	109.5
N1—C7—H7	120.8	H16A—C16—H16B	109.5
C1—C7—H7	120.8	O4—C16—H16C	109.5
O2—C8—N2	120.4 (2)	H16A—C16—H16C	109.5
O2—C8—C9	122.0 (2)	H16B—C16—H16C	109.5
N2—C8—C9	117.7 (2)		

### Hydrogen-bond geometry (Å, °)

**Table:**

*D*—H···*A*	*D*—H	H···*A*	*D*···*A*	*D*—H···*A*
N2—H2···O4^i^	0.899 (10)	1.997 (12)	2.881 (3)	167 (3)
O4—H4···O2	0.82	2.35	2.989 (3)	135
O4—H4···O2^ii^	0.82	2.34	3.023 (3)	141
O1—H1···N1	0.82	1.84	2.557 (3)	145

Symmetry codes: (i) −*x*, −*y*+1, −*z*+1; (ii) −*x*+1, −*y*+1, −*z*+1.
